# Prevalence of partnerships between bacteria and ciliates in oxygen-depleted marine water columns

**DOI:** 10.3389/fmicb.2012.00341

**Published:** 2012-09-19

**Authors:** William Orsi, Sophie Charvet, Peter Vd'ačný, Joan M. Bernhard, Virginia P. Edgcomb

**Affiliations:** ^1^Department of Geology and Geophysics, Woods Hole Oceanographic InstitutionWoods Hole, MA, USA; ^2^Département de Biologie, Université LavalQC, Canada; ^3^Department of Zoology, Comenius University, Mlynská dolina B-1Bratislava, Slovak Republic

**Keywords:** ciliate, SEM, rRNA, anoxic, OMZ, hypersaline, symbiosis, CARD-FISH

## Abstract

Symbioses between Bacteria, Archaea, and Eukarya in deep-sea marine environments represent a means for eukaryotes to exploit otherwise inhospitable habitats. Such symbioses are abundant in many low-oxygen benthic marine environments, where the majority of microbial eukaryotes contain prokaryotic symbionts. Here, we present evidence suggesting that in certain oxygen-depleted marine water-column habitats, the majority of microbial eukaryotes are also associated with prokaryotic cells. Ciliates (protists) associated with bacteria were found to be the dominant eukaryotic morphotype in the haloclines of two different deep-sea hypersaline anoxic basins (DHABs) in the Eastern Mediterranean Sea. These findings are compared to associations between ciliates and bacteria documented from the permanently anoxic waters of the Cariaco Basin (Caribbean Sea). The dominance of ciliates exhibiting epibiotic bacteria across three different oxygen-depleted marine water column habitats suggests that such partnerships confer a fitness advantage for ciliates in these environments.

## Introduction

Over the last decade, a growing number of molecular surveys have demonstrated a high diversity of ciliates in oxygen-depleted and anoxic marine water-column environments (Alexander et al., 2009; Stoeck et al., 2009; Behnke et al., 2010; Edgcomb et al., 2011b; Orsi et al., 2012b). These works revealed the presence of many uncultured ciliate lineages distantly related to cultured representatives known to harbor symbiotic prokaryotic partners. Recently, efforts have been made to visualize and describe the cellular morphologies of novel ciliate clades (Orsi et al., 2012a), and their functional roles within ecosystems (Orsi et al., 2012b).

Ciliates exhibit diverse symbioses with bacteria (e.g., Bernhard et al., 2000; Edgcomb et al., 2011a; reviewed in Fenchel and Finlay, 1995 and Gast et al., 2009). In most anaerobic ciliates, the evolution of hydrogenosomes from mitochondria may reflect their adaptation to an anaerobic lifestyle (Embley, 2006). Hydrogenosomes produce ATP and hydrogen through the activity of the hydrogenase enzyme, and, as a result, many hydrogenosome-bearing ciliates acquired endosymbiotic methanogenic archaea capable of reducing CO_2_ with H_2_. This association between the eukaryotic host and methanogen is mutualistic because the functioning of hydrogenase (produced by the ciliate) requires a low partial pressure of H_2_, which is facilitated by the methanogen (van Hoek et al., 2000). Protists with the ability to adapt to anaerobic and sulfidic habitats would have less competition for resources (particulate organic matter and attached or free-living bacteria and archaea) compared to other eukaryotes maladapted to such conditions. Indeed in benthic marine habitats, most protists inhabiting anoxic and suboxic substrates exhibit symbioses with prokaryotic partners (Bernhard et al., 2000). Here, we report that ciliates with associated epibiotic bacteria dominate the eukaryotic communities within three different oxygen-depleted marine water-column environments: the Cariaco Basin (Caribbean Sea, Venezuela), Discovery Basin (Mediterranean Sea, Greece), and Urania Basin (Mediterranean Sea, Greece). The Urania and Discovery Basins contain brine lakes of supersaturated (~5 M) sodium and magnesium chloride, respectively (van der Wielen et al., 2005). Both basins exhibit a halocline and have detectable levels of sulfide, up to 16 mM in the Urania brine (van der Wielen et al., 2005). The combination of sulfide and hypersalinity make the Urania and Discovery basins some of the most polyextreme habitats on Earth. Our observation of abundant eukaryotic-prokaryotic partnerships suggests that such associations provide a fitness advantage for microbial eukaryotes in oxygen-depleted, hypersaline, and anoxic/sulfidic marine water column habitats.

## Materials and methods

### Sample collection

Samples from the Cariaco Basin were collected in May of 2008 (Edgcomb et al., 2011c) located at 11° 30′ N, 65° 40′ W off the north coast of Venezuela. As described in Edgcomb et al. (2011b), sampling was conducted aboard the B/O *Hermano Gínes*, operated by Estación de Investigaciones Marinas (EDIMAR), Fundación la Salle de Ciencias Naturales, located on Margarita Island, Venezuela. Samples for scanning electron microscopy (SEM) were collected from anoxic waters (900 m depth) using the DEEP-SID In Situ Sampler (Taylor and Doherty, 1990; Taylor et al., 1993; Taylor and Howes, 1994). The DEEP-SID sample collection chambers were prefilled before deployment with a mixture of Bouin's fixative and glutaraldehyde. Volumes used provided a final concentration of 0.2% glutaraldehyde and 50% Bouin's fluid. The DEEP-SID was programmed on deck to collect and fix in situ, a 4 L sample of water. Fixed samples were transferred on deck to a carboy, stored at 4°C and processed within 24 h at the EDIMAR shore lab on Margarita Island.

Samples from Urania (35° 14′ N, 21° 29′ E) and Discovery (35° 19′ N, 21° 42′ E) basins were collected in July 2009 and December 2011 on board the R/V *Oceanus* and R/V *Atlantis*, respectively. Water samples from the halocline of both basins (3500 m water depth) were acquired with a Niskin rosette equipped with a conductivity-temperature-density scanner (CTD) (Sea-Bird Electronics, Bellevue, WA, USA). Samples were fixed with 2% glutaraldehyde (final concentration) immediately after being brought on board, and stored at 4°C for 12 h before processing. The salinity of the halocline seawater was determined using a high range CTD (Neil Brown Ocean Sensors, Inc.) and confirmed using a refractometer. Sample hydrochemistry is presented in Table 1.

**Table 1:** **Physicochemical data for sampling sites at Discovery, Urania, and Caricao Basin**.

**Sample**	**Coordinates**		**Depth (m)**	**Total salinity PSU**	**Oxygen ml/L**	**Conductivity (S/m)**	**Sulfide mM**
Discovery reference	35°19.248N	21°41.462E	3578	38.74	1.69	4.75	b.l.d.
Discovery halocline	35°19.248N	21°41.462E	3580	70.01	0–0.50	7.10	n.d.
Discovery brine	35°19.248N	21°41.462E	3582	95.70 (4990 mM Mg^2+^)	b.l.d.	11.33	0.7 (see Lin et al., 2006)
Urania halocline	35°13.674N	21°28.583E	3467	63.22	0–1.22	7.80	n.d.
Urania brine	35°13.674N	21°28.583E	3472	>99.00 (3500 mM Na^+^)	b.l.d.	15.60	16 (see Lin et al., 2006)
Cariaco Basin station A	10.50°N	64.66°W	900	36.2 (see Edgcomb et al., 2011c)	b.l.d.	n.d.	0.05 (see Edgcomb et al., 2011c)

b.l.d., beyond level of detection; n.d., not determined.

### Enumeration of total protists

Total protistan cells were enumerated from Urania and Discovery halocline samples using a standard protocol for staining protistan nuclei with DAPI (Massana et al., 2006). In short, halocline water was fixed with 2% formaldehyde (final concentration) and filtered onto a 0.2 μm polycarbonate filter (Millipore). Filters were washed twice with 10 ml sterile water and air-dried. Filter sections were cut off each filter and ~20 μl of 0.2% metaphor agarose was pipetted onto the filter section. Filter sections were incubated in sterile 0.5 ml microcentrifuge tubes with 500 μl of DAPI (2 μg/ml) in the dark for 5 min. Filter sections were washed for 2.5 min in 70% ethanol, followed by 2.5 min in 100% ethanol, and air-dried. DAPI stained protists were visualized and enumerated using an Axioplan2 epifluorescence microscope (Zeiss, Germany). Total protistan cells were enumerated from the Cariaco Basin anoxic waters at 900 m depth using SEM (Edgcomb et al., 2011b). Counts of ciliates exhibiting visible epibonts from the three locations were analyzed in a Multi-Response Permutation Procedure (MRPP) to identify a statistically significant effect of oxygen concentration on their distribution. MRPP analysis was performed in PC-ORD (MjM Software Design).

### SEM preparation and enumeration of ciliates

We followed the method for SEM as described in Stoeck et al. (2003). In short, fixed samples were filtered onto 0.4 μm polycarbonate Transwell membrane filters (Corning, USA) and washed with 1X PBS (pH 7.4). Transwells were then taken through dehydration series in preparation for SEM and fixed with 100% hexamethyldisilizane (Electron Microscopy Sciences, Hatfield, Pennsylvania) before air-drying. It was critical not to expose the Transwell filters to air at any point during the protocol, until this final step, as this causes most fixed protists to collapse. The entire procedure was completed within 24 h after sampling and the air-dried Transwell filters were wrapped in aluminum foil and shipped back to the United States at room temperature. For SEM observation, filters were attached to a carbon adhesive tab and mounted on a SEM specimen holder. Mounted specimens were then sputter coated with 10–15 nm of gold and palladium (60:40) using a Tousimis Samsputter 2A and visualized with a Hitachi S4800 scanning electron microscope. A minimum of 50 microscopic fields (0.5 × 1.0 mm) was observed to count ciliates in each of the samples, and ciliates were counted on at least three different filters. A minimum of 10 specimens from each sample were used for the morphological analysis and length to body width calculations.

### Catalyzed reporter deposition—fluorescent *In situ* hybridization (CARD-FISH)

Samples from Urania and Discovery, collected in 2009 and 2011 respectively, were analyzed with CARD-FISH using the general bacterial probe EUBI-III (Daims et al., 2001), the subclass specific probes DELTA495abc and competitor cDELTA495abc (Lücker et al., 2007), ESP549 (Lin et al., 2006), GAM42a (Manz et al., 1992) and a non-sense probe NON338 (Wallner et al., 1993) for a negative control. Our protocol followed a modified version of Pernthaler et al. (2002), which was described thoroughly in Edgcomb et al. (2011a). Briefly, filter sections were prepared for hybridization by first embedding the cells with 0.2% Metaphor agarose and drying at 46°C, then inactivating the endogenous peroxidases by submerging filters 10 min in 0.01 M HCl. Permeabilization of bacterial cells was conducted in a lysozyme solution (10 mg/mL Lysozyme; 0.05 M EDTA; 0.1 M Tris-HCl, pH 8) at 37°C for 60 min, followed by washing in deionized sterile water and absolute ethanol. The hybridization was carried out at 46°C for 2.5 h in a 300:1 mix of Hybridization Buffer (0.36 M NaCl; 8 mM Tris-HCl, pH 8; 40 mg/mL dextran sulfate; 35% formamide; 0.4% Roche Blocking Reagent; 0.08% SDS) and HRP-conjugate probes EUBI-III or NON338 (working solutions 50 ng/μL; Biomers, Ulm, Germany). For DELTA495abc, EPS549 and GAM42a the hybridization was carried out at 35°C for 5–6 h in a 100:1 mix of Hybridization Buffer (35% formamide, except for the EPS549 probe which was done at 55% formamide). From this point forward, the filter sections hybridized with the different probes were treated separately. Subsequently, filter sections were rinsed in washing buffer (5 mM EDTA; 20 mM Tris-HCl, pH8; 70 mM NaCl; 0.01% SDS) for 5 min at 48°C, then incubated 15 min at room temperature in PBS 1X, to equilibrate the probe-delivered HRP. For signal amplification with catalyzed reporter deposition, filter sections were then incubated in the dark for 15 min at 37°C in a mix of amplification buffer (1X PBS, 0.1% Roche Blocking Reagent, 2 M NaCl and 0.1 g/mL dextran sulfate), H_2_O_2_ (0.015%) and the fluorescently labeled tyramide, Alexa488. This was followed by two washing steps in 1X PBS, one wash in deionized sterile water, and one wash in absolute ethanol, after which the filter sections were left to dry, and mounted onto microscope slides with a drop of DAPI-Citifluor-VectaShield (1 μg/mL DAPI; 1571 μl Citifluor; 286 μL VectaShield; 143 μL PBS 1X). Slides were then observed under epifluorescence, using a Zeiss Axio Imager M2 microscope equipped with a Zeiss AxioCam camera (Carl Zeiss Microscopy GmbH, Germany). Ciliates were detected under DAPI-excitation fluorescence (350 nm), and CARD-FISH targeted bacterial cells were visualized under GFP-excitation (500 nm) epifluorescence.

## Results

### Cariaco basin

**Figure 1:**
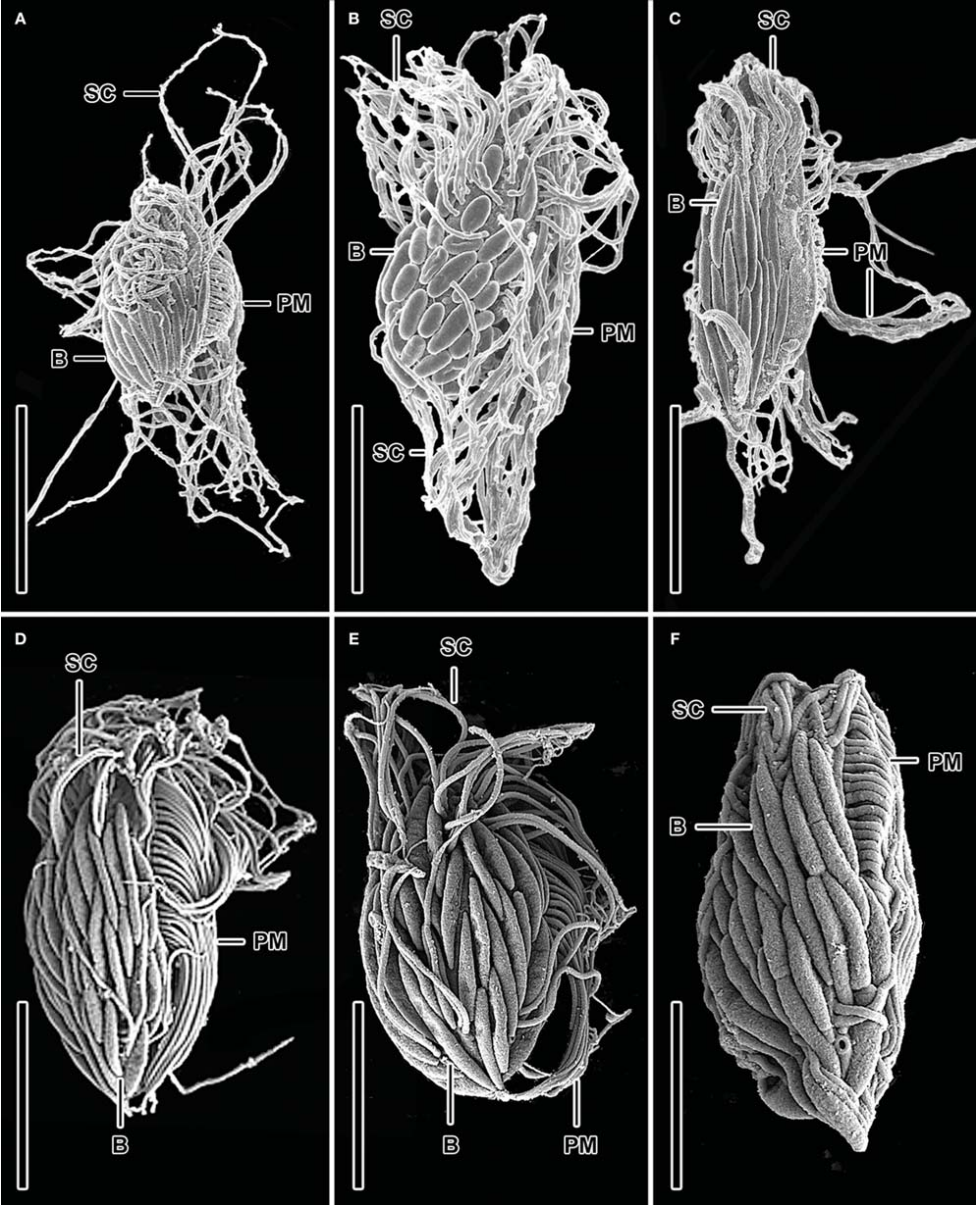
**SEM micrographs of various bacteria/ciliate morphologies from the Cariaco Basin in the Caribbean Sea (A–C) and the halocline of the Urania Basin in the Mediterranean Sea (D–F).** Dorsolateral **(A, B, D–F)** and dorsal **(C)** views of possible scuticociliates exhibiting epibiotic Bacteria which are usually localized on the posterior two-thirds or four-fifths of the unciliated dorsal side of the ciliates' body. Explanations: B = Bacteria; PM = paroral membrane; SC = somatic cilia. Scale bars: **(A)** = 5 μm; **(B)** = 9 μm; **(C)** = 7 μm; **(D–F)** = 3 μm. Images **(A–C)** are reformatted from (Edgcomb et al., 2011b).

Enumeration of total protists from the Cariaco Basin revealed total protist cell numbers of approximately 3 × 10^5^ cells/liter (Edgcomb et al., 2011b; Table 2). SEM observations of samples from the anoxic waters of the Cariaco Basin revealed abundant ciliates associated with epibiotic bacteria or archaea (Figures 1A–C) (see refs Edgcomb et al., 2011b; Orsi et al., 2012a for discussion). Ciliates were present at approximately 10^4^/L (based on observation of 500 fields of view on 25 filters each representing 50 ml) (Table 2). Some ciliates resemble scuticociliates (Scuticociliatia, Oligohymenophorea) in having a prominent paroral membrane extending over half of the body length as well as one to several caudal cilia (Figures 1A–C), while others correspond to a novel ciliate class, the Cariacotrichea (Orsi et al., 2012a). The paroral membrane (frequently referred to as the paroral) is a compound ciliary organelle lying along the right side of the oral area. The scuticociliate-like type has paroral cilia forming a prominent tuft that usually extends far posteriorly behind the body. The 1.5–5 μm-long epibiotic prokaryotic partners of these scuticociliate-like organisms are usually localized on the posterior two-thirds or four-fifths of the unciliated dorsal side of the ciliates' body (Figures 1A–C). Over 90% of ciliates observed in anoxic samples from the Cariaco Basin had visible epibiotic microbes, whereas no such partnerships were observed in oxygenated waters above the oxycline.

**Table 2:** **The abundance of two ciliate morphotypes containing epibiotic bacteria compared to total protistan cells**.

	**Urania halocline**	**Discovery halocline**	**Cariaco basin**
Ciliates with visible epibonts (10^4^ cells L^−1^)	0.97 (±0.2)	1.16 (±0.2)	1.4 (±0.7)
Total protistan cells (10^4^ cells L^−1^)	1.82 (±0.6)	1.98 (±0.25)	31.2 (±0.2)

**Figure 2:**
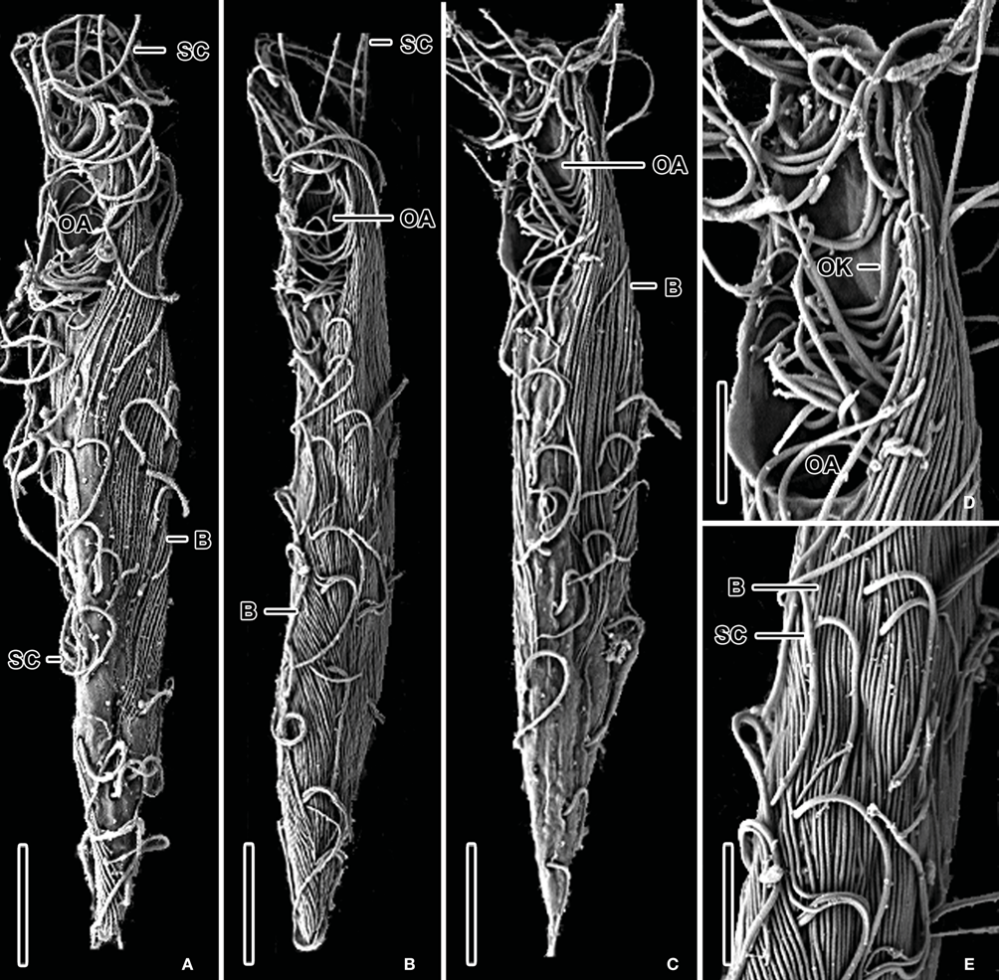
**SEM micrographs of various bacteria/ciliate morphologies from the halocline of the Discovery basin in the Mediterranean Sea.** Ventrolateral views **(A–C)** of narrowly fusiform ciliates displaying epibiotic filamentous bacteria which cover almost the whole ciliates' body. Detail of oral apparatus **(D)** of a specimen shown in **(C)**. There is a single oral kinety, which could be a reduced or modified adoral organelle, on the left corner of the oral cavity. Surface view **(E)** showing 10–20 μm-long, slightly sigmoidal, filamentous Bacteria attached to the ciliates' cortex. Explanations: B = Bacteria; OA = oral apparatus; OK = oral kinety; PM = paroral membrane; SC = somatic cilia. Scale bars: **(A,C)** = 10 μm; **(B)** = 12 μm; **(E)** = 6 μm; **(D)** = 4 μm.

**Figure 3:**
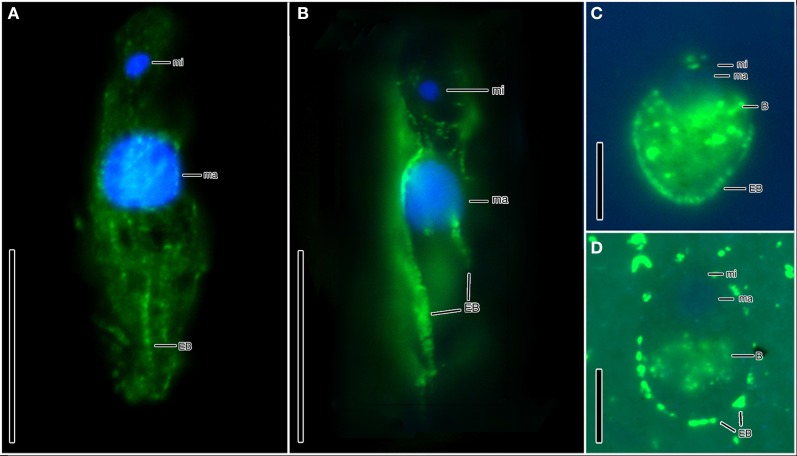
**Hybridization of CARD-FISH probes to bacterial epibonts attached to ciliates from Discovery (A,B) and Urania (C,D) haloclines. (A)** Epifluorescence of the EUB I-III CARD-FISH probe confirming that the microbes attached to the cortex of a ciliate from Discovery halocline are Bacteria. **(B)** Epifluorescence of a CARD-FISH probe specific to Delta-proteobacteria, confirming that the microbes attached to the cortex ciliates from Discovery halocline are Delta-proteobacteria. **(C,D)** Epifluorescence of the EUB I-III bacterial probe hybridized with epibiotic microbes attached to the cortex (as well as internal microbes) of the scuticociliate morphotypes in Urania halocline. Explanations: ma = macronucleus; mi = micronucleus; EB = epibiotic bacteria; B = bacteria (either ingested prey or endosymbionts). Scale bars: **(A,B)** = 30 μm; **(C,D)** = 10 μm.

The abundance of two ciliate morphotypes present in Urania (see Figures 1D–F and 3C,D) and Discovery (see Figures 2A–E and 3A,B) haloclines, the Cariaco Basin (see Figures 1A–C), and total protists as determined by DAPI enumeration.

### Urania halocline

Ciliates similar to the scuticociliate-type observed in Cariaco are present at a concentration of 9.7 (±0.2) × 10^4^ cells L^−1^ in the Urania halocline (Figures 1D–F). These ciliates also have a distinct paroral membrane extending at least half of the body length and their paroral cilia usually form a long tuft extending in the posterior direction. Similar to that observed in Cariaco, the epibionts of these scuticociliate-like organisms are 2–2.5 μm-long and are localized on the same region of the ciliates' body (Figures 1D–F). CARD-FISH analyses confirm that the epibiotic cells attached to the cortex of these ciliates are bacteria (Figures 3C,D). Bacterial cells were also observed within the cytoplasm (Figures 3C,D), corresponding to either internal symbionts or ingested prey. This abundant scuticociliate morphotype dominates the Urania halocline, representing 95% of all ciliate morphotypes observed. Partnerships between ciliates and bacteria were not observed in oxygenated waters above the halocline or in the anoxic brine. A comparison to the abundance of total protists enumerated with DAPI reveals that this scuticociliate-like morphotype is the most abundant eukaryotic lifeform (representing >50% of total eukaryotic cells) in the Urania halocline (Table 2).

### Discovery halocline

The halocline of Discovery Basin contains an abundance of ciliates of similar morphology (Figures 2A–C,E) that are present at a concentration of 3.7 (±0.3) × 10^5^ cells L^−1^. These ciliates are narrowly fusiform and contain an oral apparatus that occupies only the anterior body fifth or fourth (Figures 2A–D). There is no paroral membrane recognizable on the right corner of the oral cavity, but on the left corner we could locate a single paroral membrane-like kinety, which could be a reduced and modified adoral organelle (Figure 2D). This structure sinks posteriorly into the oral cavity, i.e., does not continue as an ordinary somatic kinety. All ciliates observed in the Discovery halocline fit this general morphological description. Most (80%) of these ciliates exhibit 10–20 μm-long, slightly sigmoidal bacterial cells attached to their cortex (Figures 2A–C,E). CARD-FISH analyses confirm that the epibiotic cells attached to the cortex of these ciliates are Delta-proteobacteria (Figure 3B). Such partnerships between ciliates and bacteria were not observed in oxygenated waters above the halocline, and no discernible protist was observed in the brine. A comparison to the abundance of total protists enumerated with DAPI reveals that this ciliate morphotype accounts for the majority (>50%) of protists present in the Discovery halocline (Table 2).

MRPP analysis of the distribution of ciliates exhibiting epibonts from Urania Halocline, Discovery Halocline, and anoxic Cariaco Basin reveals that oxygen concentration has a statistically significant correlation with their distributions (*p* = 0.02).

## Discussion

### Ciliate morphotypes in hypersaline and/or oxygen-depleted marine water columns

SEM observations revealed that hypersaline and oxygen-depleted deep ocean basins harbor at least three ciliate morphotypes. Within the sulfidic Cariaco Basin, we detected (among other morphotypes) a completely new cytoarchitectural type of ciliate, which was classified into a distinct class Cariacotrichea (Orsi et al., 2012a), and a scuticociliate-like cell type (Figures 1A–C), which very likely belongs to the class Oligohymenophorea. A strikingly similar scuticociliate-like morphotype was also observed within the halocline of the hypersulfidic and hypersaline Urania basin (Figures 1D–F). However, a different ciliate morphology associated with different bacteria was dominant within the halocline of Discovery basin (Figure 2), an environment characterized by high concentrations of magnesium chloride and relatively lower sulfide (Table 1). The scuticociliate-morphotype (Figures 1D–E) was not observed in the Discovery Basin and the Discovery morphotype (Figure 2) was not observed in the Cariaco and Urania basins. Thus, these different morphotypes appear restricted to waters, each with a specific hydrochemistry. The dominance of scuticociliate morphotypes is consistent with reports of relatively high numbers of scuticociliate-affiliated 18S rRNA genes recovered from other anoxic marine habitats, such as the Saanich Inlet and Framvaren Fjord (Orsi et al., 2012b). Our observations are supported by a statistical analysis of the distributions of ciliate morphotypes exhibiting epibonts. This test reveals oxygen concentration explains a significant portion of these ciliates distributions.

Ciliates in the three environments we sampled fall into two dominant morphotypes. As noted, within the Cariaco and Urania basins, the ciliates have an ellipsoidal to bluntly fusiform body with a length/width ratio of approximately 2.5:1. Their oral apparatus occupies the anterior third or half of the body, and is equipped with a prominent paroral on the right corner of the oral cavity or with an archway kinety delineating anterior as well as the left and right margin of the oral cavity. The morphology indicates that these ciliates are potentially effective bacterial grazers in deep-sea oxygen-depleted waters. This is consistent with the observation of bacterial cells within the cytoplasm (Figures 3C,D), that are most likely ingested prey contained inside of food vacuoles (or possibly endosymbiotic bacteria). On the other hand, ciliates from the Discovery Basin are very narrowly fusiform in shape, having a length/width ratio of about 8:1. Their oral apparatus is distinctly shorter, occupying only one fifth or fourth of the body length, and the ciliature appears to be reduced as we could locate only a single kinety on the left corner of the oral cavity. The reduction of oral ciliature may reflect a different feeding strategy than the scuticociliate-like morphotypes seen in Urania and Cariaco that exhibit a row of oral cilia, usually extending over half the body length. An alternative hypothesis is that the Discovery halocline ciliates rely less on heterotrophic grazing and more on their putative symbionts for nutrition. Such a possibility is corroborated by observations of a karyorelictean ciliate, *Kentrophoros fistulosus*, which displays only oral vestiges and is completely dependent on its epibiotic bacteria for nutrition (Foissner, 1995). A nutritional basis for symbioses between ciliates and bacteria has been documented for at least five different species of ciliates, and is most common between ciliates and ectobiotic (as opposed to endobiotic) bacteria (reviewed in Gast et al., 2009). The microbial epibonts that *K. fistulosus* is dependent upon for nutrition are sulfate-reducing bacteria (Gast et al., 2009). The relationship between the Discovery ciliate morphotype and its bacterial epibonts may have a similar basis, as these epibonts are Delta-proteobacteria (Figure 3B), a group that contains many lineages of sulfate-reducing bacteria.

It is important to note that there may be some relationship between body length/width ratio of ciliates and their epibiotic bacteria. Specifically, comparatively broader ciliate and bacterial morphotypes (length/width ratio <8:1) dominate in the anoxic Cariaco water column and Urania halocline water column, while more slender morphotypes (length/width ratio 8:1) dominate in the Discovery halocline water column. This morphological trend suggests hypersulfidic environments select for broader morphologies, while perhaps magnesium chloride environments foster a narrower body shape. Replication and dedicated study of additional high magnesium chloride habitats are required to confirm this possible explanation.

The unique hydrochemistry of each basin studied has been shown to select for distinct prokaryotic assemblages (van der Wielen et al., 2005; Daffonchio et al., 2006; Taylor et al., 2006; Lin et al., 2007; van der Wielen and Heijs, 2007). These differences in prokaryotic communities may have indirectly led to the different partnerships between eukaryotes and prokaryotes that we observed (Figures 1 and 2). Thus, it is likely that the similar hydrochemistry of Cariaco and Urania Basins (dominant salt cation Na^+^, hypersulfidic) has selected for a similar abundant scuticociliate/epibont morphotype observed in both locations (Figure 1). Our collective observations to date highlight the strong selective nature of geochemistry on marine microbial distributions.

### Eukaryote-prokaryote partnerships are diagnostic of oxygen-depleted marine waters

Associations between protists and prokaryotic partners in marine sedimentary oxyclines have been noted previously (e.g., Bernhard et al., 2000; Edgcomb et al., 2011a). Our observation of abundant ciliates with putative symbionts in three different water-column oxyclines support previous observations that partnerships between ciliates and bacteria are a common feature of oxygen-depleted habitats (e.g., Fenchel and Finlay, 1995; Gast et al., 2009). Molecular surveys of microbial eukaryotes in oxygen-depleted habitats have detected an abundance of novel ciliate phylotypes affiliating with scuticociliates and other oligohymenophorean ciliates (Edgcomb et al., 2011b; Orsi et al., 2012b), corroborating our SEM observations of these ciliate types and the MRPP test for effect of oxygen concentration on their distributions (*p* = 0.02). The recent discovery of a new class of ciliates, the Cariacotrichea (Orsi et al., 2012a), whose representatives are associated with epibiotic bacteria and are restricted to anoxic marine habitats, further demonstrates that we still have much to learn about protists, and interactions between protists and prokaryotes in oxygen-depleted and anoxic marine waters. The high abundance of ciliates associated with epibiotic bacteria in these environments, relative to total protistan cells (Table 2), suggests that this partnership confers a fitness advantage in halocline and/or oxycline environments. Given the global trend of decreasing dissolved oxygen concentrations in the world's oceans (e.g., Keeling et al., 2010), we predict that such partnerships will become more prevalent as marine oxygen minimum zones continue to expand.

### Conflict of interest statement

The authors declare that the research was conducted in the absence of any commercial or financial relationships that could be construed as a potential conflict of interest.
